# Case Report: Coronaro-bronchial fistula vascularizing a squamous cell lung cancer

**DOI:** 10.3389/fcvm.2023.1279611

**Published:** 2023-11-08

**Authors:** Chloe Ntshaykolo, Thomas Fave, Clement Benic, Antoine Boizet, Margaux Geier, Pierre-Philippe Nicol, Renaud Descourt

**Affiliations:** Centre Hospitalier Universitaire (CHU) de Brest, Hôpital Morvan, Département d'Oncologie Thoracique, Brest, France

**Keywords:** coronary fistula, coronaro-bronchial fistula, lung neoplasms, coronaropathy, immunotherapy

## Abstract

Coronary fistulas are rare, having been described for the first time by Krauss in 1865 in postmortem. They are commonly asymptomatic and can be caused by congenital or acquired malformations. We present the case of a 65-year-old patient who was treated for squamous cell lung cancer with chemoimmunotherapy and presented with angina. The coronary angiography showed a coronaro-bronchial fistula that arises from a branch of the right coronary artery and is associated with lung cancer.

## Introduction

Initially described by Krauss in 1865 in postmortem, a coronary fistula is an abnormal precapillary communication of a coronary artery or one of its branches into a cardiac cavity or other low-pressure vascular structure (1, 2). Coronary fistulas are a rare event with an incidence between 0.2% and 0.6% per year (3). Coronaro-cameral fistulas are more frequent in pediatric populations (congenital), whereas coronary–pulmonary fistulas are more frequent in adults on a process that seems acquired (1).

Squamous cell lung cancer is the second most common type of non-small cell lung cancer, which represents the first cause of cancer death in men and the second in women.

The present case reports a case of a patient with coronaro-bronchial fistula, incidentally discovered during an acute cardiac episode, vascularizing the bronchial tumor in the patient followed up since December 2020 for lung carcinoma.

## Case presentation

**Figure 1:**
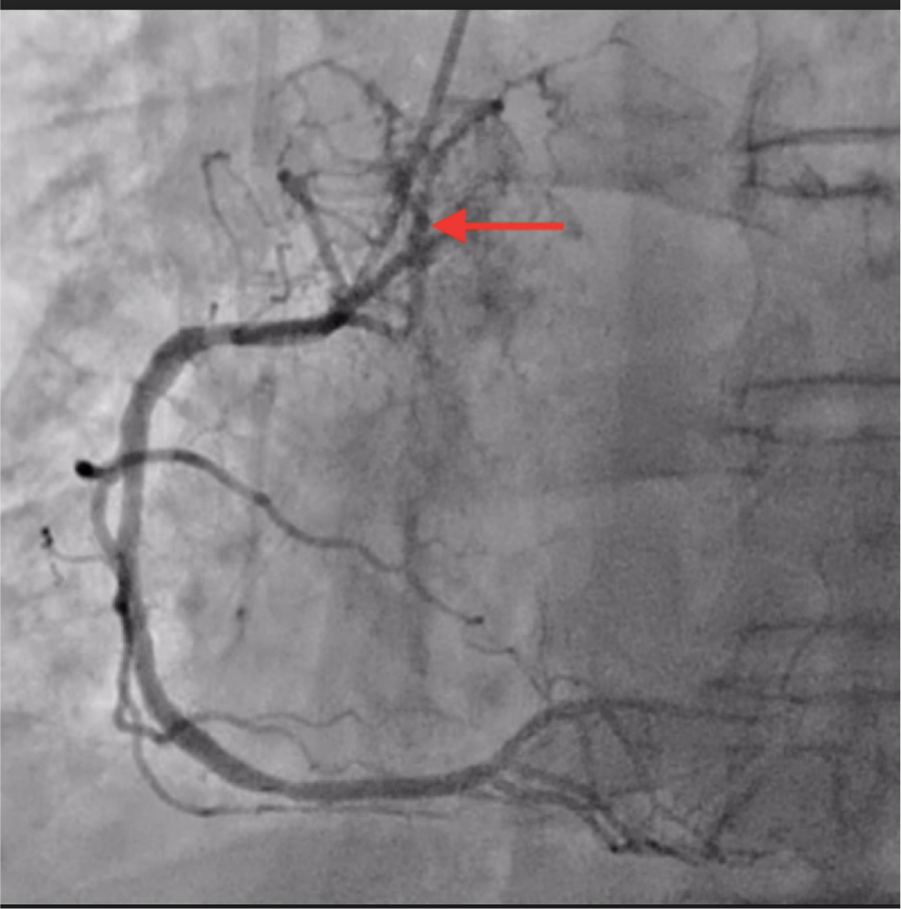
Fistula origin (coronary angiography).

A 65-year-old Caucasian man, an active smoker of 30 pack-year, was diagnosed with an advanced squamous cell lung cancer cT4N2M1a (contralateral lung nodules). Programmed death-ligand 1 (PDL1) tumor proportion score (TPS) was between 1% and 5% in December 2020. He started chemoimmunotherapy (paclitaxel–carboplatin–pembrolizumab) in January 2021 followed by pembrolizumab maintenance because of a partial tumor response. While on pembrolizumab, he presented to the emergency unit in December 2021 with left chest pain. ECG and troponin assays (initial troponin 1,490 ng/L and then 19,300 ng/L) led to the diagnosis of an ST-troponin + acute coronary syndrome. Further explorations were carried out urgently:

- Cardiac echography showed an apical and apico-septal hypokinesis, overflowing in the middle third of the anteroseptal wall of the left ventricle. Left ventricular ejection fraction (LVEF) and cardiac output were preserved. The right ventricle was normal in size and function.
- Coronary angiography highlighted a right dominance, tandem significant stenosis first segment of left anterior descending artery with ostial significant stenosis diagonal of small caliber with kinetics appearing altered in territory first segment of left anterior descending artery. Significant stenosis bisecting with a poorly developed downstream bed. An atypical coronary fistula was noted, arising from a branch of right coronary artery and perfusing the bronchial tumor (coronaro-bronchial fistula) (Figure 1).

Anterior interventricular artery stenting following percutaneous transluminal coronary angioplasty was performed. After discussions with interventional radiologists and the referring oncologist, embolization of the coronaro-bronchial fistula was not performed because this procedure seemed risky for the coronary artery system.

**Figure 2:**
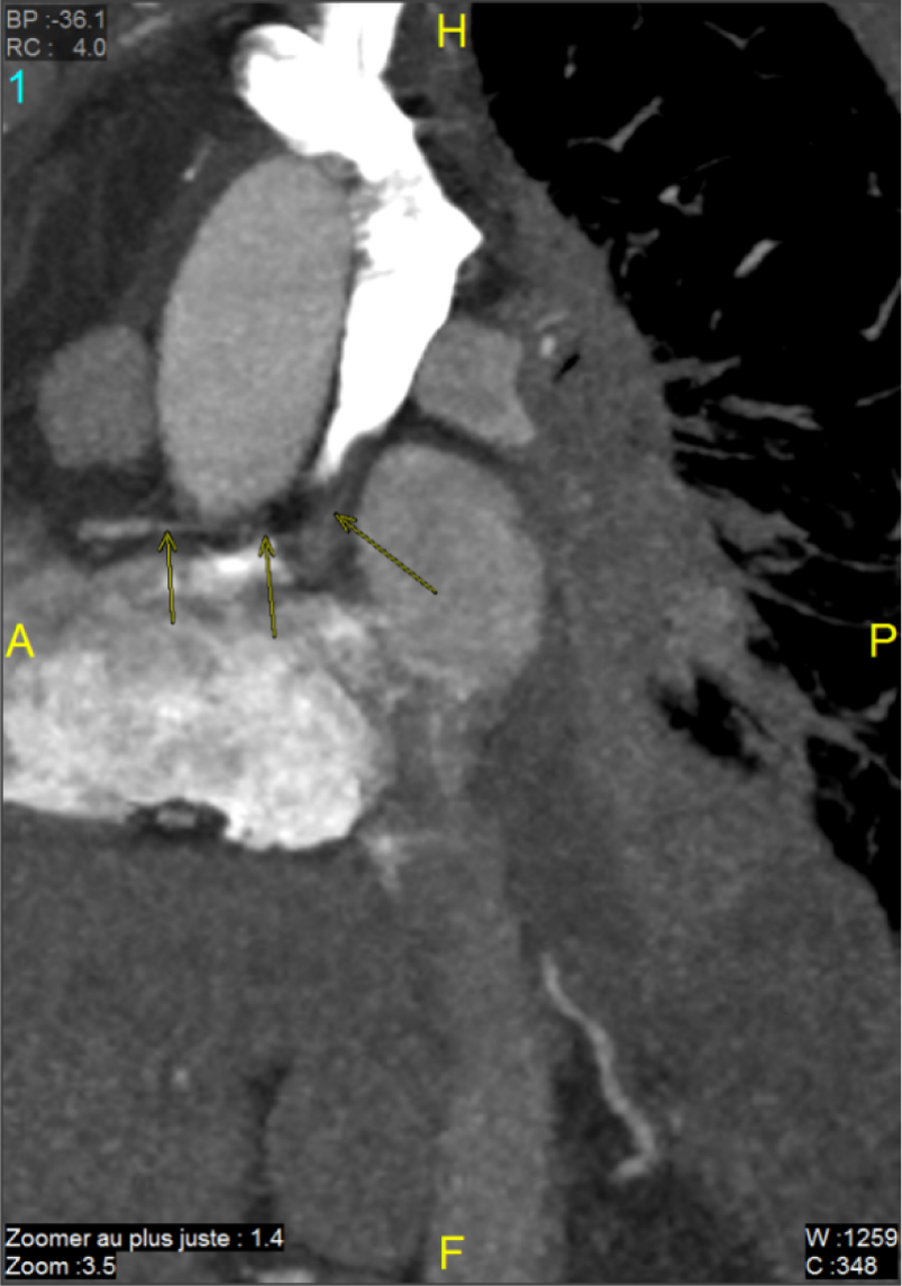
Sagittal section: the path of the ectopic bronchial artery (coroscan).

**Figure 3:**
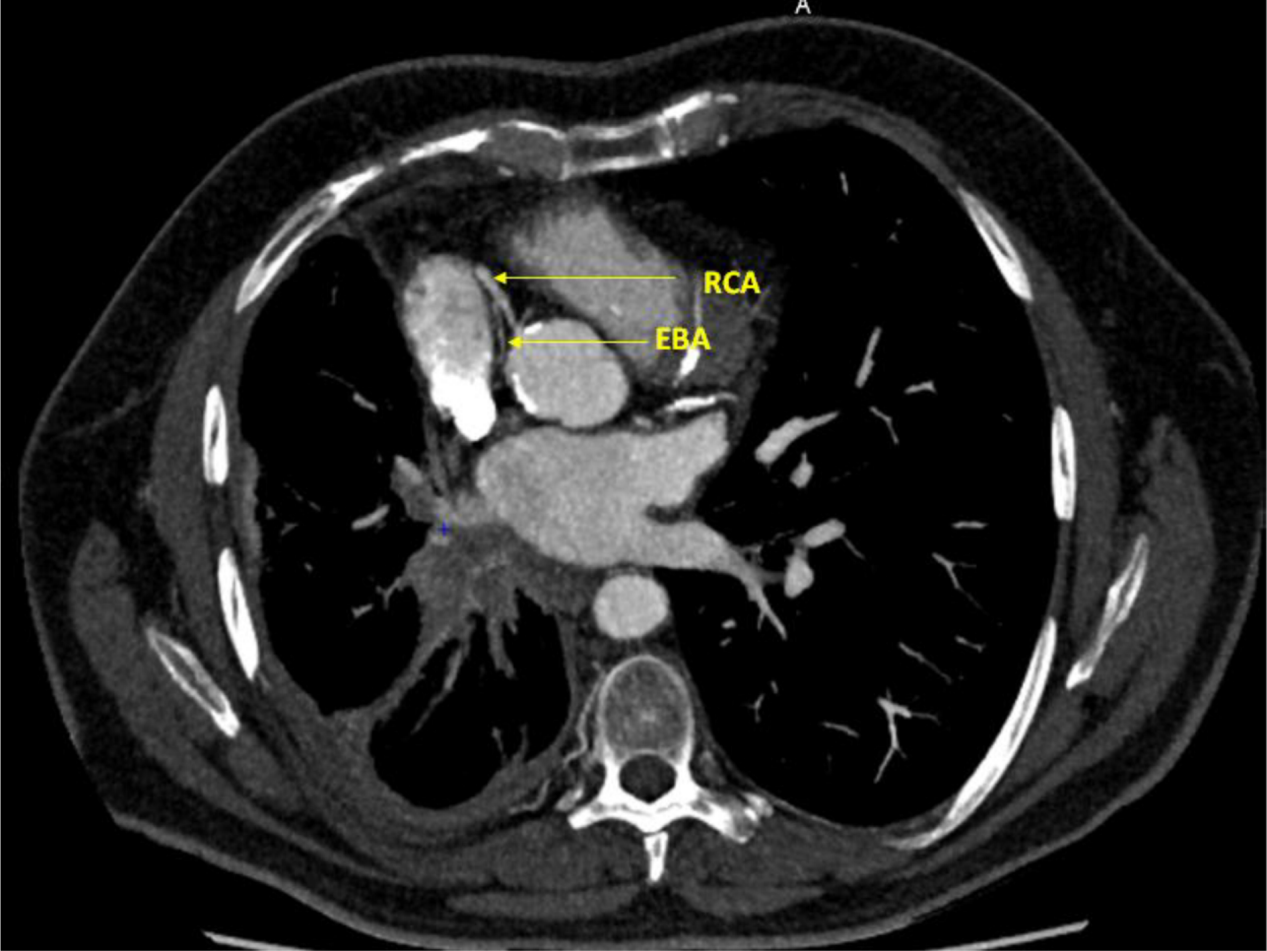
Axial section, right coronary artery (RCA) and ectopic bronchial artery (EBA) (coroscan).

Immunotherapy was continued. The latest tumor assessment in January 2022 showed a stable disease. We asked the radiologists to perform a CT scan to determine the site of the fistula (Figures 2, 3).

## Discussion

Coronary fistulas are rare malformations arising more often from the right coronary artery (RCA). Most of them are congenital, but some have been demonstrated to be iatrogenic after cardiac surgery by some case reports (4).

The genesis of a coronary arterial fistula is the result of communication between a coronary artery, a cardiac chamber, a large vessel, or any other vascular structure, initially described by Krauss in 1865 in postmortem (5). It is an abnormal connection between the arteries and a vascular structure of low pressure. In most cases, coronary fistulas are asymptomatic and diagnosed incidentally. In older patients, certain signs can be seen, such as exertional dyspnea, exertional angina, arrhythmia, endocarditis, and stroke (1, 6).

For this patient, the possible causes of this fistula are neoangiogenesis of his cancer and predisposing conditions, age, male sex, and consumption of an estimated 60 packs of weaned tobacco per year.

We consider that this is not a toxicity of immunotherapy, because we have never found similar cases in the literature. However, cardiac toxicities occur, and there is a relationship between immunotherapy and myocardial infarction: 2% of myocardial infarctions are reported with pembrolizumab, and the mechanisms are:

- Associated inflammation may enhance plaque rupture, which is the main mechanism in the case of our patient.
- Systemic inflammatory response providing coronary spasm.
- Direct T cell-mediated.

The reference diagnostic examination remains coronary angiography, which studies the anatomy of the fistula. Cardiac Doppler ultrasound, especially transesophageal, and cardiac CT scans provide additional morphological information, such as coronary artery affected, diameter of the feeder artery, and diagnosis of complications (1).

The complications of coronary fistulas can be severe including hypoperfusion of the adjacent myocardium, thrombosis, embolism, heart failure, atrial fibrillation, rupture, endocarditis or endarteritis, and arrhythmia (1).

Regarding the different therapeutics, there are two possible approaches, namely, surgical or percutaneous closure (7). Surgical treatment has shown excellent efficacy and long-term safety. Percutaneous closure is also a reliable method with good results (8). In more than 70% of the cases, metallic coils, balloons, and umbrella-type systems rarely covered stents (6, 9).

The emergency intervention would have been embolization (e.g., by a coil) through the fistula to stop bleeding from the tumor.

Given the necrotic nature of the tumor, the risk of fistula rupture is likely, but we did not apply any specific follow-up. The patient underwent frequent CT scans as part of his neoplastic follow-up. We could have realized coroscanner scans, but the cardiologists maintained a standard annual follow-up. Finally, the coronagrophy and the fistula remained unchanged.

Shao et al. (9) reported a case of a coronary fistula and a lung adenocarcinoma, incidentally discovered in a 67-year-old woman admitted to the hospital for a persistent cough. The CT scan found an isolated nodule in the upper lobe of the left lung. Transthoracic echocardiography as part of a preoperative workup showed the coronary fistula confirmed later at angiography but without a link between them. In this case, they adopted a combined operation and surgical treatment of both conditions in a single procedure via sternotomy.

Our case shows a real “coronaro-tumoral” fistula, arising from the RCA and perfusing the lung tumor.

In this patient who was followed up in 2020 and is in good general condition, PS 0-1, the question of treatment arises.

The therapeutic indications remain a predominant question, there is no consensus. In adults, spontaneous closure is rare, and it requires elective surgical or percutaneous closure by “coils” (10).

The patient was eligible for chemotherapy because the benefit–risk ratio was discussed at the multidisciplinary team, and the prognosis was related to neoplasia.

At first, the neoplasia was a locally advanced stage, but radiochemotherapy was not practicable in light of the excessively wide fields of irradiation, so it was considered stage IV. The expected and declared OS, according to KEYNOTE-407, is 18–24 months.

In the neoplastic context, the situation is more complex and will require multidisciplinary consultation, taking into account several elements, disease evolution, oncological prognosis, the pathophysiological impact of the fistula, and the general condition of the patient.

Indeed, no literature review on this case has supported the decision taken.

## Conclusion

This is the first case of an active coronary bronchial fistula vascularizing a pulmonary neoplasm in a patient with good general condition. It was decided to perform a simple surveillance without embolization due to the limited experience with this anomaly and its rarity.

## Data Availability

The original contributions presented in the study are included in the article/Supplementary Material, further inquiries can be directed to the corresponding authors.
